# IL-6 production through repression of *UBASH3A* gene via epigenetic dysregulation of super-enhancer in CD4^+^ T cells in rheumatoid arthritis

**DOI:** 10.1186/s41232-022-00231-9

**Published:** 2022-11-03

**Authors:** Kaoru Yamagata, Shingo Nakayamada, Tong Zhang, Anh Phuong Nguyen, Naoyuki Ohkubo, Shigeru Iwata, Shigeaki Kato, Yoshiya Tanaka

**Affiliations:** 1 The First Department of Internal Medicine, University of Occupational and Environmental Health, Japan, 1-1 Iseigaoka, Yahata-nishi, Kitakyushu, Fukuoka, 807-8555 Japan; 2 Graduate School of Life Science and Engineering, Iryo Sosei University, Iwaki, Fukushima, 970-8551, Japan

**Keywords:** Rheumatoid arthritis, CD4, Super-enhancer, UBASH3A, IL-6

## Abstract

**Background:**

Rheumatoid arthritis (RA) is associated with immune dysfunction. UBASH3A as a negative regulator of T cell receptors (TCRs) signaling is a susceptible factor in RA. The aim of this study was to determine the role of UBASH3A in RA pathogenesis, by assessing the role of super-enhancer (SE) in the control of UBASH3A expression in CD4^+^ T cells and the contribution of the latter in proinflammatory cytokine production in patients with RA.

**Methods:**

UBASH3A mRNA and protein levels were quantified by PCR and western blotting, respectively. The cells were treated with a locked nucleic acid to inhibit enhancer RNA (eRNA) expression. Chromatin immunoprecipitation was used to identify the factors recruited to *UBASH3A* loci displaying SE architecture. CD4^+^ T cells were transfected with *UBASH3A* plasmids, and cytokine levels were measured by a cytometric bead array.

**Results:**

*UBASH3A* was extracted as a RA susceptibility gene associated with SNPs in the SEs that are highly expressed in CD4^+^ T cells by in silico screening. UBASH3A mRNA and protein expression levels were lower in CD4^+^ T cells of RA patients than in the control. eRNA_1 and eRNA_3 knockdown reduced *UBASH3A* mRNA levels. RA patients exhibited accumulation of BTB and CNC homology 2 (BACH2), the silencing transcription factor, at the *UBASH3A* loci in CD4^+^ T cells, but not the SE-defining factor, mediator complex subunit 1 (MED1)/bromodomain 4 (BRD4). However, opposite changes were observed in the control. Stimulation of TCRs expressed on CD4^+^ T cells of RA patients resulted in interleukin (IL)-6 production, while UBASH3A over-expression significantly inhibited the production.

**Conclusions:**

In RA, transcription of *UBASH3A* is suppressed via epigenetic regulation of SE in CD4^+^ T cells. Low UBASH3A levels result in excessive TCR signal activation with subsequent enhancement of IL-6 production.

**Supplementary Information:**

The online version contains supplementary material available at 10.1186/s41232-022-00231-9.

## Background

Rheumatoid arthritis (RA) is a systemic autoimmune disease characterized by synovitis and progressive joint destruction [1–3]. The pathogenesis of RA includes overexpression of proinflammatory cytokines, such as tumor necrosis factor (TNF)-α, interleukin (IL)-6, IL-1β, and matrix metalloproteinase 3, in the synovial fluid, and lymphocyte infiltration in the synovial membrane [4, 5]. Ectopic overexpression of IL-6 is a critical factor that induces several RA-related pathoimmune processes, such as imbalances between T helper 17 (Th17) cells and regulatory T cells (Tregs), overproduction of autoimmune antibodies, systemic inflammation, and joint destruction [4]. Reflecting the roles of these factors in RA pathology, their inhibitors have been used therapeutically in clinical practice [6]. Although some patients experience RA remission following the use of such inhibitors, others fail to achieve remission or experience relapse, suggesting that the identification of RA drivers remains limited in terms of the pathogenesis and progression of RA.

A set of such unidentified RA drivers appears to be associated with the patient’s genetic background because 101 single-nucleotide polymorphisms (SNPs) for susceptibility gene loci have already been identified by genome-wide association studies (GWASs) in large cohorts [7]. Most SNPs related to any disease are generally located in the non-coding regions instead of the protein-coding mRNA gene loci. Importantly, most of the RA-susceptibility SNPs have been mapped to enhancer regions. Compared with typical enhancers, the super-enhancers (SEs), which are large enhancer clusters consisting of individual enhancers, harbor 3.2 times more RA-susceptibility SNPs, suggesting that RA-susceptibility SNPs are strongly associated with transcriptional regulation via SEs [8, 9]. Strikingly, 26% (27/101) of the RA-susceptibility SNPs are located within the activated SEs in CD4^+^ T cells [10]. SEs serve as pivotal enhancers to define cell identity through directing global gene regulation. The function of SEs has been recently delineated to induce dynamic chromatin looping between the SE regions and the promoter regions of the target genes with the aid of enhancer RNAs (eRNAs) transcribed from the SE regions.

Taking into consideration the previous findings of RA-associated SNP mapping in SEs, we reasoned that SE dysfunction is causal for RA progression by dysregulating the expression of RA-related gene(s) directed by the SE. To test this hypothesis, we examined CD4^+^ T cells consisting of several subsets of helper T (Th) cells because CD4^+^ T cells serve as drivers in RA pathogenesis by producing inflammatory cytokines. Using the in silico approach with multiple databases, we identified a RA-susceptibility gene using our gene mining criteria. This gene encodes ubiquitin-associated and SH3 domain-containing protein A (UBASH3A), which has been reported to attenuate T cell receptor (TCR) signaling by targeting the transforming growth factor β (TGF-β)-activated kinase 1/nuclear factor-kappa B (NF-κB) signaling axis. Since the RA-associated SNP (rs189352) has been reported in a SE of the *UBASH3A* locus [7], we also assessed the expression of UBASH3A and its regulatory mechanism in CD4^+^ T cells, and found that UBASH3A expression was indeed downregulated in RA CD4^+^ T cells, presumably due to dysfunction of the *UBASH3A* SE.

## Methods

### In silico study (bioinformatic tools)

dbSUPER (https://asntech.org/dbsuper/) is a database that describes 82,234 SEs in 102 human and 25 mouse tissue/cell types. RA-susceptibility genes were detected by GWAS, as reported previously [11]. BioGPS (http://biogps.org/#goto=welcome) is a database that summarizes the patterns of mRNA expression specific to human and mouse tissues and cells that have been identified by oligonucleotide array analysis.

### Preparation of CD4^+^ T cells

CD4^+^ T cells were prepared as described previously [2]. First, CD4^+^ T cells were collected from peripheral blood mononuclear cells (PBMC) of 11 healthy donors [male to female=3:8; mean age, 42.5±7.3 (±SD) years] and of 24 RA patients (8:16; 49.3±5.1 years, respectively), with clinical disease activity index (CDAI) of 136±49, simplified disease activity index for rheumatoid arthritis (SDAI) 140±51, and disease activity score-28 (DAS28) of 6.1±1.2. The T cells were prepared using CD4^+^ microbeads of the CD4^+^ T Cell Isolation kit (#130-096-533; Miltenyi Biotec, Auburn, CA) (Supplementary Table 1). CD4^+^ T cells were sorted into subsets by fluorescence-activated cell sorting (FACS) gating. Cells were then seeded onto six-well plates at a density of 1 × 10^6^ cells/well and incubated in Roswell Park Memorial Institute (RPMI) 1640 medium containing 10% fetal bovine serum and 1× antibiotics at 37°C under 5% CO_2_ atmosphere. The positive control Jurkat cells were treated with anti-CD28 (PV1) or anti-CD3 (2C11) for 6 h to activate TCR signaling [12].

### Immunoblotting

CD4^+^ T cells were prepared from PBMC of 3 healthy donors (male to female 1:2) and 3 patients with RA (male to female 1:2). We also used CD4^+^ T cell line Jurkat as a positive control. Immunoblotting was performed as described previously [13]. Briefly, whole-cell extracts were prepared from lysis of PBMC in TNE buffer (50 mM Tris-hydrochloride [pH 7.4], 0.5% [v/v] Nonidet P-40, 150 mM sodium chloride, 5 mM ethylenediaminetetraacetic acid, 50 mM NaF, 1 mM sodium orthovanadate, 1 mM phenylmethylsulfonyl fluoride, 10 mg/mL leupeptin, and 10 mg/mL aprotinin). Proteins (7 μg) were separated by sodium dodecyl sulfate-polyacrylamide gel electrophoresis and transferred onto nitrocellulose membranes (Protran BA83; GE Healthcare, Chalfont St. Giles, UK) for blocking. The membranes were incubated with anti-phospho-NF-κB antibody (Ser 536, #3033; Cell Signaling Technologies, Danvers, MA), anti-NF-κB antibody (#8242; Cell Signaling Technologies), anti-UBASH3A antibody (GTX116432; GeneTex; Irvine, CA), and anti-β actin antibody (A1978; Sigma-Aldrich, St. Louis, MO). The bound antibodies were visualized with secondary antibodies against mouse and rabbit immunoglobulin G (IgG, GE Healthcare) conjugated with horseradish peroxidase and chemiluminescence reagent (ECL™ Prime western blotting Detection Reagent; GE Healthcare).

### Cytometric bead array (CBA)

CBA was performed as described previously [13]. Briefly, the supernatant from cultured CD4^+^ T cells of 3 RA patients (male to female 1:2) and 3 healthy donors (male to female 1:2) was collected 24 h after transfection of the cells with plasmid cloning DNA (pcDNA) 3.1-UBASH3A (courtesy of Professor Patrick Concannon) and empty vector. Next, 20 μL of the supernatant was reacted with 0.2 μL of capture beads of the Human Flex Set (#558279 for IL-1 beta, #558276 for IL-6, #560383 for IL-17A, and #558273 for TNF; BD Biosciences, San Diego, CA) and 19.8 μL of RPMI medium for 1 h at room temperature. Then, 0.2 μL of phycoerythrin detection reagent and 19.8 μL of RPMI medium were added, and the mixture was allowed to react for 1 h. The supernatant was rinsed twice with flow cytometry staining (FACS) buffer, centrifuged, and suspended in 150 μL of FACS buffer. Finally, each cytokine was detected by FACS, and its concentration was analyzed by FCAP Array software.

### Reverse transcription-quantitative polymerase chain reaction (RT-qPCR)

RT-qPCR was performed as described previously [14]. Briefly, the total RNA was extracted from cultured CD4^+^ T cells of 3 RA patients (male to female 1:2) and 3 healthy donors (male to female 1:2) using the RNeasy Mini kit (Qiagen, Hilden, Germany) and reverse-transcribed using SuperScript®VILO™ Master Mix (Life Technologies). On a Step One Plus system (Applied Biosystems), qPCR was performed with TaqMan®Fast Universal PCR Master Mix (Applied Biosystems) and the TaqMan® Gene Expression Assay (Applied Biosystems) primer/probe pairs to measure the expression of the following genes (Supplementary Table 2): *UBASH3A*, interleukin-1 beta (*IL-1β*), *IL-6*, *IL-17A*, tumor necrosis factor-alpha (*TNF-α*), and glyceraldehyde-3-phosphate dehydrogenase (*GAPDH*).

### Histopathological examination

A single patient with RA and a single patient with dermatomyositis, who met the diagnostic criteria of the two conditions, consented to provide lymph node samples. This study was conducted with the approval of the ethics committee of the University of Occupational and Environmental Health. Lymph node tissue samples were collected, fixed in 10% formaldehyde, and embedded in paraffin. Immunofluorescence staining was performed as described previously [13]. Briefly, the biopsy samples were immersed in phosphate-buffered saline with Tween (PBST; 0.05% [v/v], pH 6.0) containing sodium citrate (5 mM) to inactivate antigens. The samples were blocked with a serum-free protein block (Dako, #2016-08) and reacted with polyclonal antibodies that recognize human CD4 (dilution, 1:200; #ab67001; Abcam, Cambridge, MA) and UBASH3A (dilution, 1:200; #ab197168, Abcam). The slides were rinsed with PBST and then incubated for 1 h with an anti-rabbit IgG secondary antibody labeled with fluorescein isothiocyanate and an anti-mouse IgG secondary antibody labeled with rhodamine (dilution, 1:500; DakoCytomation, Glostrup, Denmark). Nuclei were stained with 4’,6-diamidino-2-phenylindole (dilution, 1:200; Merck). The stained samples were examined under an all-in-one fluorescence microscope. The results of the staining were quantified using ImageJ software as described previously [15].

### Chromatin immunoprecipitation-PCR (ChIP-PCR)

ChIP-PCR was performed as described previously [14]. CD4^+^ T cells collected from 5 healthy donors (male to female 1:4) and 5 RA patients (male to female 1:4) were cultured in RPMI 1640 medium. Chromatin was cross-linked with 1% formaldehyde for 10 min and fragmented to 200–500 bp by sonication. DNA was extracted using the EZ ChIP Kit (Merck Millipore, Taufkirchen, Germany) using the protocol provided by the manufacturer. DNA was immunoprecipitated using a series of antibodies. PCR was performed using specific primers (Supplementary Table 2).

### Plasmid transfection

Following the instructions provided by the manufacturer, we transfected pcDNA3.1-UBASH3A and empty vector into CD4^+^ T cells (1 × 10^5^ cells) collected from 3 healthy donors (male to female 1:2) and 3 RA patients (male to female 1:2) using Lipofectamine 3000 and P3000 reagents (Thermo Fisher Scientific, Waltham, MA). In contrast, locked nucleic acid (LNA) was designed to knock down the expression of eRNA (Supplementary Table 2). LNA targeting eRNA and control LNA were transfected into CD4^+^ T cells (1 × 10^5^ cells) with RNAi MAX (Thermo Fisher Scientific) reagents for 72 h, using the protocol provided by the manufacturer.

### Genotyping

SNP-PCR was performed according to the instructions supplied by the manufacturer to determine the genotypes of rs1893592-A/C, as described previously [16]. On a Step One Plus system (Applied Biosystems), PCR was performed in 10 μL of reaction volume containing 1 μL of genomic DNA (20 ng/μL) from 14 RA patients (male to female 4:10), 5 μL of TaqMan Universal PCR Master Mix, 0.25 μL of VIC/FAM-labeled probe (200 nM), and 3.75 μL of double-distilled water. Supplementary Table 2 lists the probes used in this process. The series of reactions was analyzed using the Allelic Discrimination Sequence Detection Software (Applied Biosystems).

### Statistical analysis

All quantified data are expressed as mean ± standard deviation. Differences between the two groups were tested for statistical significance by the Student’s unpaired two-tailed *t* test or Dunnett’s multiple comparison test. Spearman’s test was used for the correlation analysis between two variables of interest. The statistical significance was set at *P*<0.05. All statistical analyses were performed using SPSS statistical software (v. 21.0; IBM Corp., Armonk, NY).

## Results

### Identification of UBASH3A as an RA-susceptibility gene by in silico mining

**Fig. 1:**
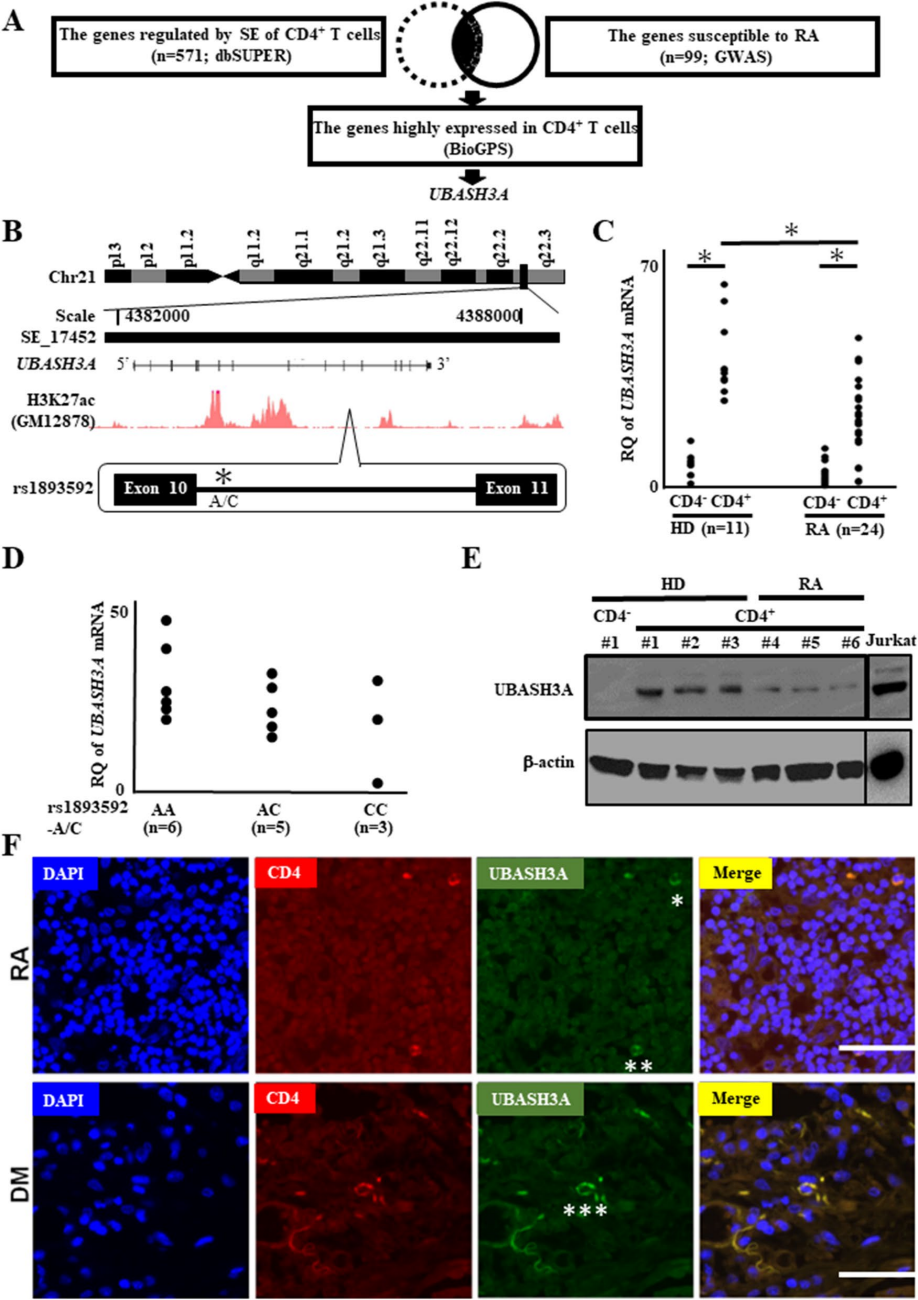
Downregulation of UBASH3A in CD4^+^ T cells of rheumatoid arthritis (RA) patients. **A** In silico screening for RA-susceptibility factors in the super-enhancer database (dbSUPER), GWAS, and biogene portal system (BioGPS). **B** Super-enhancer (SE_17452) domain mapped to the *UBASH3A* locus in CD4^+^ T cells. The locus is mapped to chromosome 21 at approximately 4,382,000–4,388,000. Histone H3 containing the acetylated lysine 27 (H3K27ac) marks are provided in GM12878, which shows lymphoblastoma cell line. Rs1893592-A/C is located in the intron between exons 10 and 11, marked with an asterisk. **C** The mRNA expression levels of the *UBASH3A* gene in CD4^−^ and CD4^+^ T cells of peripheral blood mononuclear cells (PBMCs) collected from healthy donors (HD, *n*=11) and RA patients (*n*=24) were evaluated by RT-qPCR. **D** Expression levels of *UBASH3A* mRNA among three allele groups of patients with RA (rs1893592-AA, AC, and CC). **E** Expression levels of UBASH3A protein in CD4^−^ and CD4^+^ T cells of HD (*n*=3) and RA patients (*n*=3) evaluated by Western blotting. β-actin was used as a loading control. **C**, **D** Amount of *UBASH3A* transcript expressed relative to that of *GAPDH* transcript. Data are mean ± standard deviation. Dunnett’s multiple comparison test. **F** UBASH3A protein expression in CD4^+^ T cells from the lymph nodes of dermatomyositis (DM) and RA patients evaluated by immunofluorescence staining. RQ, relative quantification. *P<0.05, by Student’s *t* test and ANOVA. Scale bar = 100 μm

Based on previous findings implicating SNPs located in SEs in RA pathogenesis, we investigated first whether dysregulated expression of RA-susceptibility genes drives RA progression. Under the concept that expression of such RA-susceptibility genes is directed by SEs harboring SNPs, we performed in silico screening for RA-susceptibility genes associated with SNPs in the SEs that are highly expressed in CD4^+^ T cells (Fig. 1A). For this purpose, *UBASH3A* was extracted using the protocol described above. The gene locus of *UBASH3A* is encompassed in a SE (designated as *UBASH3A*-SE hereafter) registered in the dbSUPER, and the SNP (rs1893592) with RA-susceptibility identified by the GWAS report [7] is positioned in this *UBASH3A* locus (Fig. 1B).

### Downregulated expression of UBSH3A in CD4^+^ T cells

In the next step, we investigated whether the expression level of *UBASH3A* was indeed dysregulated during RA progression. PBMC of RA patients and control subjects were separated into CD4^+^ T and CD4^-^ cells by FACS gating. RT-qPCR showed significantly low *UBASH3A* mRNA expression levels in CD4^+^ T cells of RA patients compared to the control (Fig. 1C). Although the SNP genotype of the rs1893592-A/C allele was reported to correlate significantly with RA susceptibility [7], we could not identify such correlation between *UBASH3A* expression and SNP genotype in our RA patients (Fig. 1D). The discrepancy in the results could be due to the limited number of subjects in the present study.

We also examined the expression of *UBASH3A* in subsets of CD4^+^ T cells, including Th1, Th2, Th17, and regulatory T cells [3]. However, no significant difference in *UBASH3A* expression was observed among the subsets (Supplementary Figure 1). To verify *UBASH3A* downregulation in RA patients, we examined USBASH3A protein levels by western blotting. The results showed reduced expression of UBASH3A protein in RA patients (Fig. 1E, Supplementary Figure 2 and Additional file 1). Furthermore, double immunofluorescence staining of CD4 and UBASH3A in CD4^+^ T cells from lymph nodes showed weaker UBASH3A staining in RA patients compared with dermatomyositis patients (Fig. 1F and Supplementary Figure 3).

### Enhancer RNAs (eRNAs) transcribed from UBASH3A-SE facilitate UBASH3A expression in CD4^+^ T cells

**Fig. 2:**
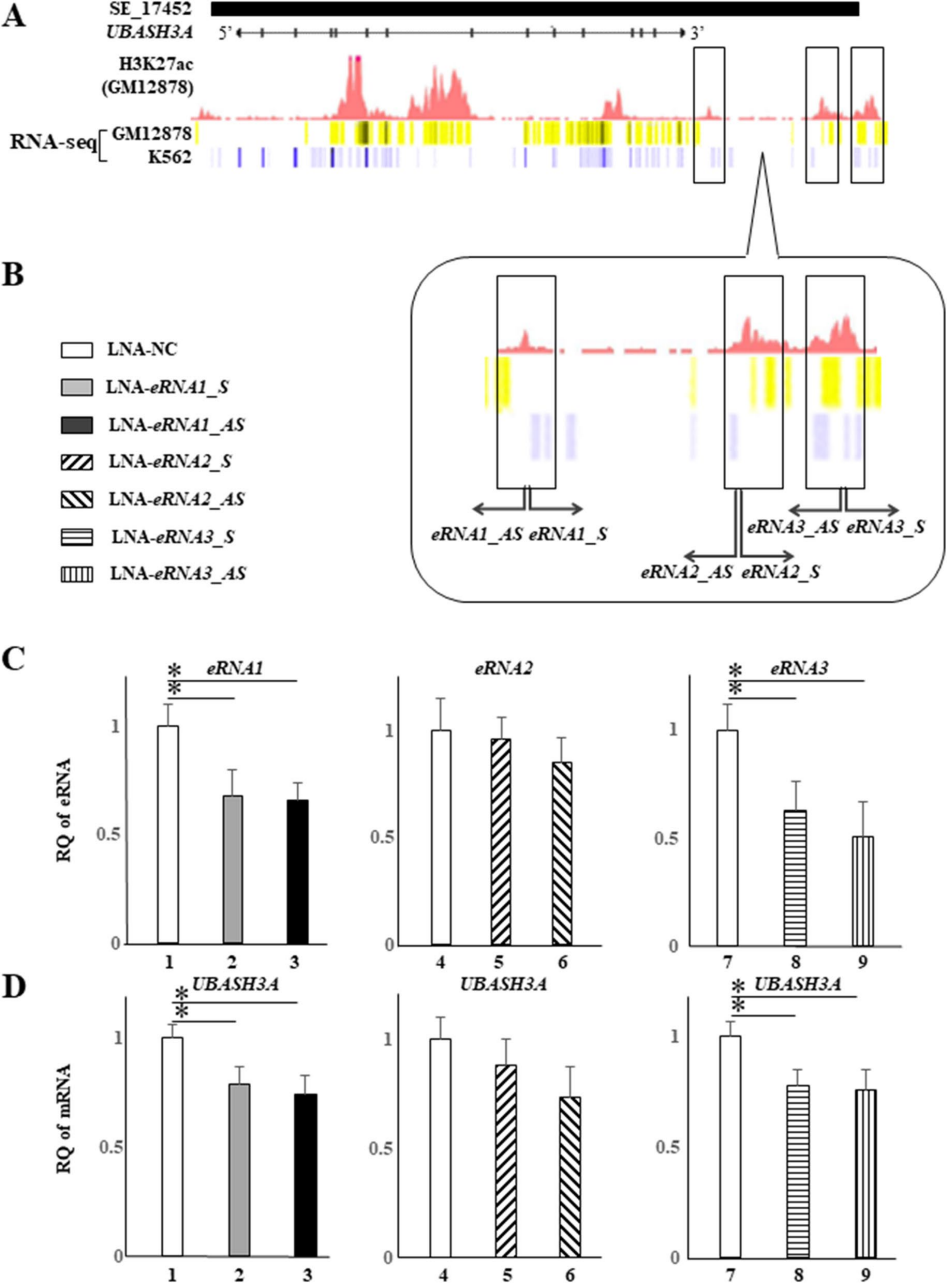
eRNA promotes *UBASH3A* transcription in CD4^+^ T cells. **A** Genomic structure of *UBASH3A*, histone H3 containing the acetylated lysine27 (H3K27ac), and transcripts peaks. Putative eRNA_1-3 are expressed within the super-enhancer (SE_17452) in CD4^+^ T cells. H3K27ac are marked in GM12878 lymphoblastoid cells. RNA-sequencing data are shown in GM12878 and K562 leukemia cells. **B** Each antisense LNA GapmeR. LNA-eRNA denotes LNA needed for eRNA knockdown. **C**, **D** Jurkat cells were transfected with LNA-NC (lanes 1, 4, and 7), LNA-eRNA1_S (lane 2), LNA-eRNA1_AS (lane 3), LNA-eRNA2_S (lane 5), LNA-eRNA2_AS (lane 6), LNA-eRNA3_S (lane 8), and LNA-eRNA3_AS (lane 9). S and AS denote sense and antisense, respectively. The amount of each transcript was quantified by RT-qPCR and then expressed relative to that of *GAPDH* transcript. Data are mean ± standard deviation of three independent experiments. *P<0.05, vs control, by Dunnett’s multiple comparison test. RQ, relative quantification

To determine the molecular basis for the observed *UBASH3A* downregulation in RA patients, we assessed the activity of *UBASH3A*-SE, because SEs are prime regions governing the transcription of adjacent genes. First, we measured the expression of a set of eRNAs (UBASH3A eRNAs) since SE activity is dependent on the expression of eRNAs transcribed from the SE. The eRNA candidates were examined by in silico screening based on H3K27ac marks documented in lymphoblastoma in the ENCODE database, as well as the registered transcript signals obtained by RNA-seq analysis of lymphoblastoma (GM12878) and leukemia cells (K562) in dbSUPER (Fig. 2A). Based on the peaks of H3K27ac, we constructed PCR primers (Fig. 2A, B) capable of detecting the expression of three transcripts (designated as *UBASH3A* eRNA_1–eRNA _3 hereafter) by qRT-PCR in CD4^+^ T cells (Fig. 2C). To assess if these eRNA candidates were functional as eRNAs that facilitate *UBASH3A* expression, we investigated the effects of their knockdown on *UBASH3A* expression using locked nucleic acid (LNA)-mediated RNAi approach (Supplementary Table 2). CD4^+^ T cells were transfected with LNAs targeting both strands of the eRNAs_1–3, and the expression levels of *UBASH3A* and eRNAs_1–3 were tested. LNAs targeting *UBASH3A* eRNA_1 and eRNA_3 attenuated their expression (Fig. 2C), but no such effect was observed with LNAs targeting *UBASH3A* eRNA_2. Reflecting the attenuated expression of eRNA_1 and eRNA_3, *UBASH3A* mRNA levels were also reduced (Fig. 2D). These findings suggest that *UBASH3A* expression is regulated, at least in part, by *UBASH3A*-SE, the regions encoding eRNA_1 and eRNA_3.

### Recruitment of silencing transcription factor to UBASH3A-SE of CD4^+^ T cells

**Fig. 3:**
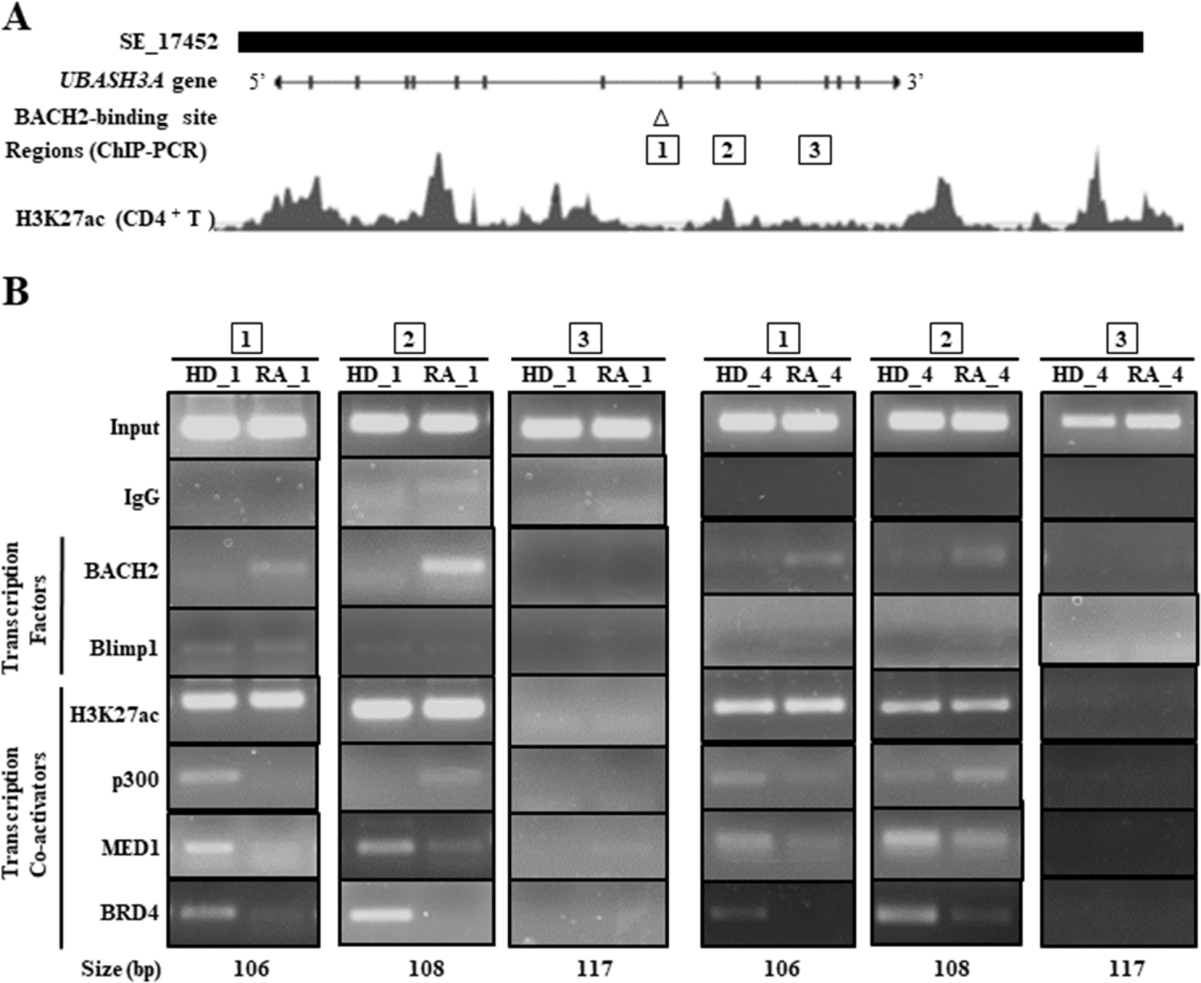
Recruitment of BACH2 to *UBASH3A* locus in CD4^+^ T cells. **A** The *UBASH3A* locus displays super-enhancer architecture. Super-enhancer (SE_17452) domain mapped in CD4^+^ T cells is located at the *UBASH3A* locus. The locus is mapped to chromosome 21 at approximately 4,382,000–4,388,000. Histone H3 containing the acetylated lysine27 (H3K27ac) marks is provided in primary CD4^+^ T cells. BACH2-binding site is mapped to the indicated *UBASH3A* locus. ChIP assay was performed with the indicated antibodies across the *UBASH3A* loci in CD4^+^ T cells of healthy donors (*n*=5; HD_1, _2, _3, _4, and _5; male to female 1:4) and rheumatoid arthritis (RA) patients (*n*=5; RA_1, _2, _3, _4, and _5; male to female 1:4). DNA regions (1–3) were amplified by ChIP-PCR. **B** Results of ChIP-PCR. ChIP-PCR was performed as described under the “Methods” section. Five independent experiments were performed with similar results. The representative data are provided as shown in HD_1 versus RA_1 and HD_4 versus RA_4

RA progression is associated with the downregulation of UBASH3A expression in CD4^+^ T cells. Accordingly, we concluded that *UBASH3A*-SE activity is compromised during RA progression. Next, we examined whether the regulatory regions encoding the UBASH3A eRNAs are dysfunctional. To investigate this issue, we explored several possible mechanisms and identified the presence of a putative binding site (TGCTGAGATGCA) for BTB and CNC homology 2 (BACH2), a silencing transcription factor, by mining the binding sites for MafK, a heterodimer partner for BACH2 (Fig. 3A) [17]. Previous studies reported overexpression of BACH2 in lymphocytes, including B and T cells [18], while mediator complex subunit 1 (MED1) and bromodomain-containing protein 4 (BRD4) are core factors underlying SE function and are detectable on the eRNA-encoding SE regions [18]. H3K27ac marks are documented in the *UBASH3A-SE* regions of CD4^+^ T cells in the ENCODE database [19]. ENCODE database shows that BRD4 and MED1 are recruited to the sites 1–3 of the *UBASH3A* gene in a variety of cell types. Thus, we addressed whether both transcriptional coactivators are recruited to the sites in the primary human CD4^+^ T cells. Consistent with the observed expression of *UBASH3A* eRNA_1 and_2, recruitment of MED1 and BRD4 was clearly observed with ChIP assay on two coding regions in CD4^+^ T cells of the control subjects. However, in CD4^+^ T cells of RA patients, this recruitment was weaker, though BACH2 recruitment was evident (Fig. 3B), suggesting reduced activity of these two regulatory SE regions. Although another transcriptional repressor (Blimp1) is expressed in T cells, its recruitment to the examined coding regions was not observed (Fig 3B and Additional files 2, 3, 4, 5). Since no clear difference in H3K27ac levels was detected between the control and RA CD4^+^ T cells, and UBASH3A was expressed at certain levels in both groups (Fig. 1C, F), we presumed that SEs were partially active at this *UBASH3A* locus.

### UBASH3A attenuates TCR signaling in CD4^+^ T cells

**Fig. 4:**
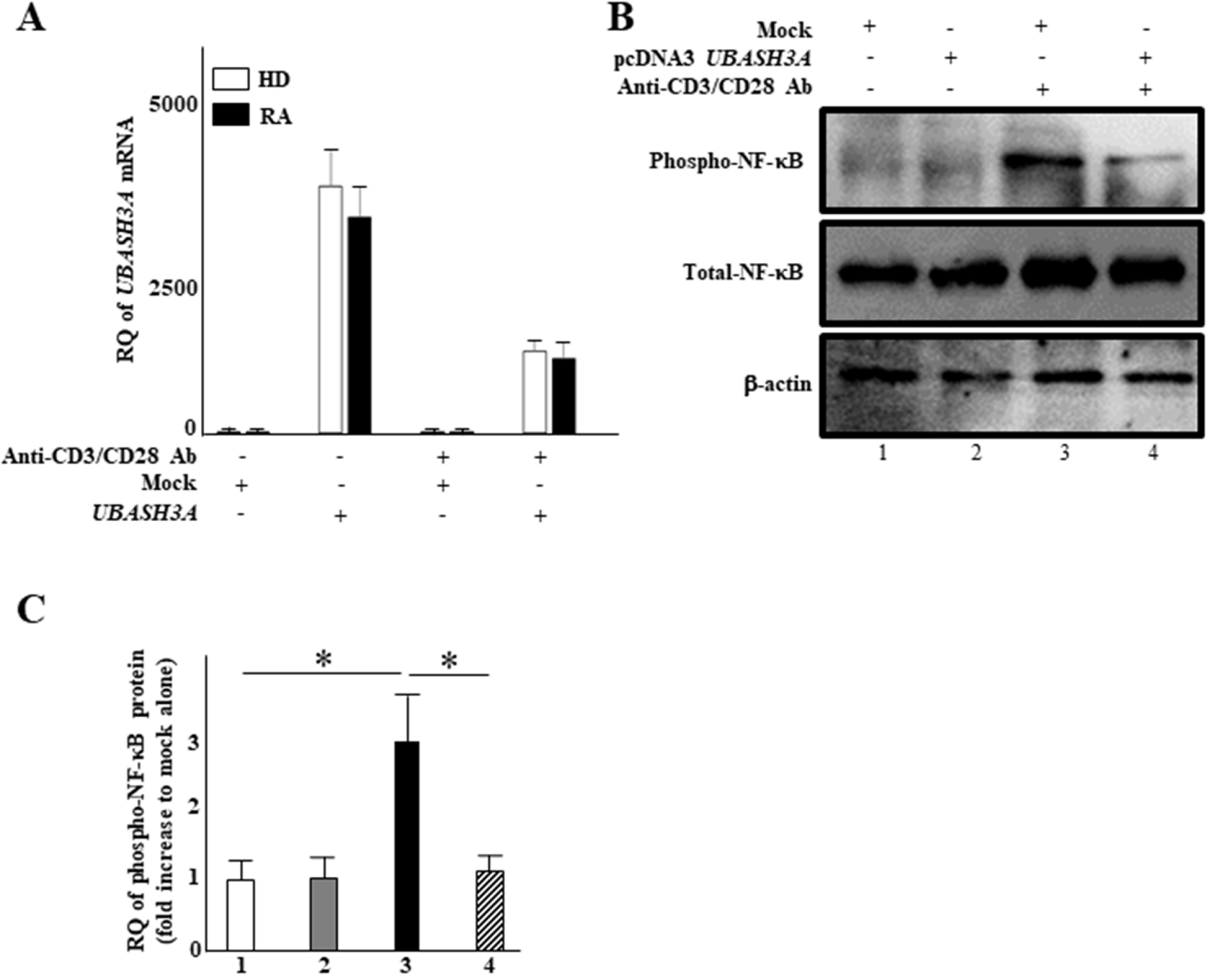
UBASH3A inhibits phosphorylation of NF-κB dependent on TCR signaling. **A**, **B** CD4^+^ T cells were transfected with pcDNA3.1-UBASH3A and empty vector. **A** At 24 h after transfection, *UBASH3A* mRNA levels were quantified by RT-qPCR. The amount of *UBASH3A* transcript was expressed relative to that of the *GAPDH* transcript. **B** At 30 min after TCR stimulation, Western blotting was performed with a series of antibodies. Data are representative of three independent Western blotting experiments. **C** The amount of total (t)-NF-κB was normalized to that of β-actin and that of phosphorylated (p)-NF-κB was then normalized to that of normalized t-NF-κB. Results were plotted on a histogram. Values are expressed as mean ± standard deviation. *P<0.05, vs lane 1 or 4, by Dunnett’s multiple comparison test. RQ, relative quantification

UBASH3A is reported to inhibit TCR signaling in CD4^+^ Jurkat cells [20]. Therefore, we assessed the inhibitory function of UBASH3A in CD4^+^ T cells. TCR signaling activation by antibodies against CD3 and CD28 [12] was monitored by phosphorylation of NF-κB, a TCR signaling downstream mediator, under the expression of UBASH3A (Fig. 4A, B and Additional file 1). Transient expression of *UBASH3A* transcript induced by its expression vector was attenuated to some extent in cells from both groups when treated with CD3 and CD28 antibodies (Fig. 4A). However, under this treatment, the induced phosphorylation of NF-κB was clearly inhibited by UBASH3A expression in CD4^+^ T cells (Fig. 4B, C), consistent with the previous reports [20].

### UBASH3A acts as a suppressor of IL-6 induction in CD4^+^ T cells

**Fig. 5:**
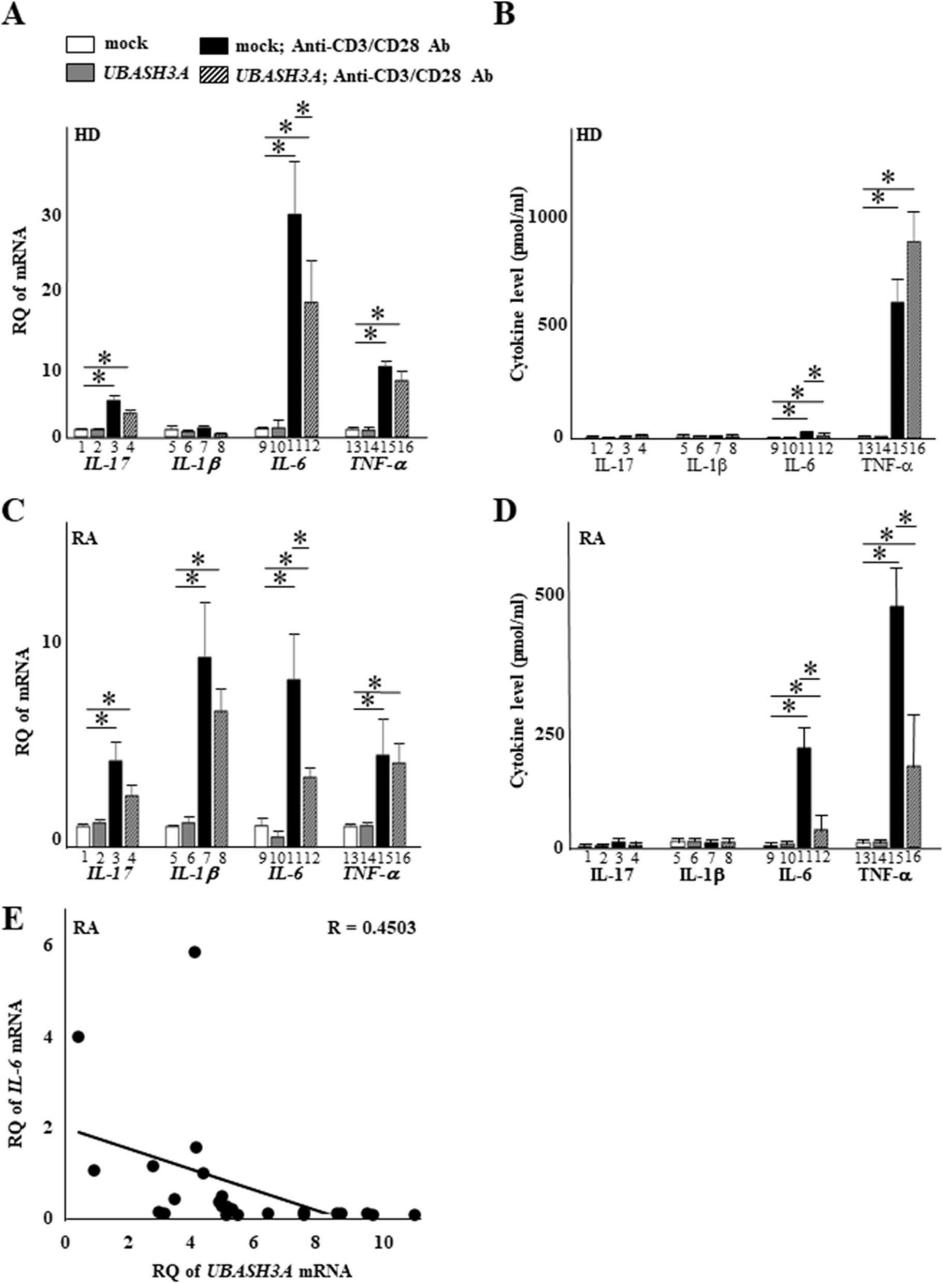
UBASH3A inhibits the production of IL-6 induced by TCR signaling in CD4^+^ T cells. **A**–**D** CD4^+^ T cells collected from healthy donors (HD) (**A**, **B**) and rheumatoid arthritis (RA) patients (**C**, **D**) were transfected with mock (lanes 1, 5, 9, and 13) and pcDNA3.1-UBASH3A (lanes 2, 6, 10, and 14) without CD3/CD28 stimulation for 24h, or mock (lanes 3, 7, 11, and 15) and pcDNA3.1-UBASH3A (lanes 4, 8, 12, and 16) with CD3/CD28 stimulation for 24h. After that, proinflammatory cytokine mRNA levels (**A** and **C**) were measured by RT-qPCR, and cytokine levels (**B** and **D**) were measured by cytometric bead array. **E** The relative mRNA expression levels of *UBASH3A* and *IL-6* were analyzed in CD4^+^ T cells of the isolated PBMCs from patients with RA (*n*=24). Spearman’s test was used for the correlation analysis between two variables of interest. The amount of cytokines transcript was expressed relative to that of *GAPDH* transcript. Data are mean ± standard deviation from three independent experiments. *P<0.05, vs mock or mock+CD3/CD28, by Dunnett’s multiple comparison test. RQ, relative quantification

Cytokines produced by T cells with activated TCR signaling play an important role in RA progression. Therefore, we investigated whether UBASH3A-attenuated TCR signaling suppresses cytokine production in TCR signaling-activated CD4^+^ T cells. Towards this end, we measured cytokines associated with RA progression in CD4^+^ T cells from healthy donors under UBASH3A-overexpressing conditions, with and without treatment with CD3 and CD28 antibodies (Fig. 5A–D). Activation of CD4^+^ T cells of the control was associated with robust production of *IL17*, *IL6*, and *TNF* mRNA (Fig. 5A) as well as IL-6 and TNF-α protein in the culture media (Fig. 5B). In CD4^+^ T cells of RA patients, augmented expression of all tested cytokines (IL-17, IL-1β, IL-6, and TNF-α) was observed as expected even without treatment (Fig. 5C). Treatment further increased the production of cytokines, particularly IL-6 and TNF-α (Fig. 5D). Most notably, overexpression of UBASH3A in CD4^+^ T cells of the control (Fig. 5A, B) and RA patients (Fig. 5C and D) suppressed the treatment-augmented cytokine production but not TNF-α protein in CD4^+^ T cells of the control, confirming the suppressive function of UBASH3A on activated TCR signaling in CD4^+^ T cells. We next aimed to investigate whether *UBASH3A* influences *IL-6* expression. We evaluated the gene expression levels of *UBASH3A* and *IL-6* in CD4^+^ T cells of PBMCs in the patients with RA. *UBASH3A* was positively correlated with the *IL-6* gene level in RA (Fig. 5E), supporting the conclusion that low levels of *UBASH3A* lead to high levels of *IL-6*.

### Recruitment of NF-κB to IL6 promoter in CD4^+^ T cells after TCR stimulation

**Fig. 6:**
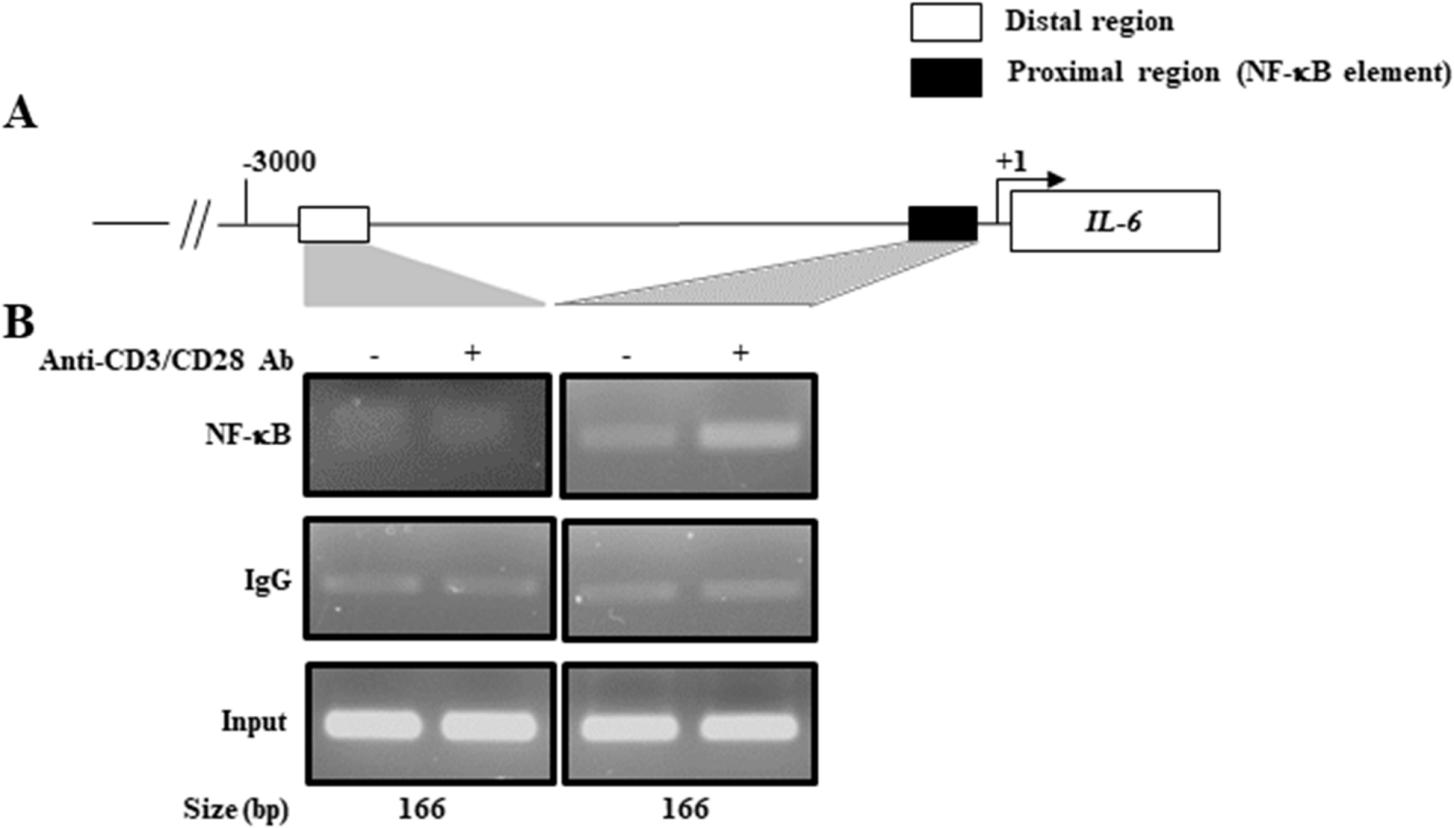
TCR signaling induces recruitment of NF-κB to *IL-6* gene promoter in CD4^+^ T cells. **A** NF-κB-binding site in *IL-6* gene promoter. **B** ChIP assay was performed with a series of antibodies in CD4^+^ T cells of healthy donors (HD) with or without TCR stimulation (30 mins). Results are representative of 3 independent experiments with similar findings

In Jurkat cells, UBASH3A suppressed IL-6 production by repressing the TCR/NF-κB pathway [21]. However, it was not clear in this study whether NF-κB directly acts on the *IL-6* promoter in primary human CD4^+^ T cells or contributes to IL-6 production as in post-transcriptional regulation without acting on the promoter. We used the DNA sequence database to predict the region of the *IL-6* promoter to which NF-κB directly binds and to elucidate the molecular mechanism of the UBASH3A-regulated NF-κB/IL-6 pathway, which we examined by the ChIP-PCR method as shown below (Fig. 6). Since CD4^+^ T cells with activated TCR signaling showed NF-κB activation, and NF-κB binding sites (GGGATTTTCC) are present in the *IL6* promoter, we performed a ChIP assay to determine whether activated NF-κB is recruited to this promoter (Fig. 6A). As shown in Fig. 6B (Additional file 6), antibody treatment resulted in clear recruitment of NF-κB at the NF-κB binding site in the region proximal to the transcription initiation site, though it was not observed at a distal region.

## Discussion

Using in silico mining, we identified *UBASH3A* as an RA-susceptibility gene, and found UBASH3A downregulation in CD4^+^ T cells of RA patients, compared with the control (Fig. 1). Consistent with previous studies, UBASH3A expression effectively attenuated activated TCR signaling in CD4^+^ T cells treated with antibodies against CD3 and CD28. Since this inhibitory effect of UBASH3A on proinflammatory cytokine production was observed in CD4^+^ T cells of both the control and RA patients (Fig. 5), it is unlikely that UBASH3A is associated with the pathogenic state of RA. As expected, UBASH3A expression inhibited NF-κB activation induced by CD3/CD28 antibody stimulation. Reflecting the induced production of proinflammatory cytokines by CD3/CD28 antibody stimulation in CD4^+^ T cells, NF-κB recruitment was more visible at the NF-κB binding site in the distal *IL6* promoter in CD4^+^ T cells following antibody treatment (Fig. 6). Since NF-κB is a major inducer of IL-6 and other proinflammatory cytokines, regulated production of these cytokines by CD4^+^ T cells appears to be, at least in part, directed by NF-κB (Fig. 5).

The presence of the RA-susceptibility SNP (rs1893592) in *UBASH3A*-SE led us to assess the SNP allelic differences in this SE function among RA patients. A case-control study using 553 patients with RA and 587 showed that a significant association between rs1893592 polymorphism and RA was found [20]. Further, a case-control study including 916 RA patients and 2266 unrelated healthy controls indicated the rs1893592 SNP in UBASH3A was related with Disease Activity Score (DAS28), C-reactive protein (CRP) level, and bone erosion [22]. The C allele of rs1893592 exhibited a pattern of negative correlation with disease activity and severity in RA patients. This allele was also reported to abnormally splice *UBASH3A* transcript in type 1 diabetes patients, thereby reducing UBASH3A protein production [21]. In contrast, in the eQTL database ImmuNexUT (https://www.immunexut.org/top), risk allele A of rs1893592 was significantly associated with decreased expression of UBASH3A in T cells [23]. However, we could not detect a clear association of this SNP allelic difference (C/A) with the expression level of UBASH3A among RA patients (Fig. 1). A larger number of patients are needed to address this issue.

SEs govern gene regulatory networks in a cell type-specific manner through the function of eRNAs transcribed from SEs. Hence, the expression of eRNAs from active SEs is considered a marker of their enhancer activity [9]. The role of eRNAs in SE function is to facilitate and stabilize dynamic chromatin looping between factors such as Yin Yang 1 (YY1) and CTCF, and the cohesion, and supportive formation of TAD structure is shared in chromatin looping events induced by eRNAs [9]. Furthermore, eRNAs are also directly associated with MED1 and BRD4, both of which are indispensable for the transcriptional initiation process [9]. More recently, chromatin looping between the SE regions and related gene promoter regions was demonstrated to form via the liquid–liquid phase separation state [9]. During the initial stages, eRNAs seem to collect various proteins required for transcription in a liquid droplet. Using the in silico to assess the intergenic regions on *UBASH3A*-SE based on H3K27ac and transcript peaks registered in the databases, we identified *UBASH3A* eRNA_1–3, as well as their expression in CD4^+^ T cells of healthy donors by RT-qPCR (Fig. 2). Our results showed that *UBASH3A*-SE transcribed eRNA_1–3 (Fig. 2), suggesting that the *UBASH3A* locus is functional as a SE. This was further confirmed by the reduction in *UBASH3A* expression by LNA-mediated knockdown of each of the tested UBASH3A eRNAs. Because LNAs for each strand of the *UBASH3A* eRNA-coding regions were effective in knocking down the expression of eRNA_1–3 as well as *UBASH3A* transcripts, the eRNA-encoding regions on *UBASH3A*-SE seem to bidirectionally transcribe sense- and anti-sense eRNAs (Fig. 2), similar to the previously characterized eRNAs on other SEs [9].

We identified UBASH3A downregulation in CD4^+^ T cells, which allowed us to speculate that *UBASH3A*-SE is dysfunctional in the RA pathogenic state. To address this issue, we investigated whether the coding regions 1–3 on *UBASH3A*-SE were functional by examining the recruitment of the factors associated with eRNAs by ChIP-PCR assay. As shown in Fig. 3B, MED1, a critical subunit in the mediator complex, and BRD4 were clearly detectable with p300 (a histone acetyltransferase acting as a transcriptional co-activator) on *UBASH3A* coding regions 1 and 2 in CD4^+^ T cells of healthy donors. However, the same was not observed on the coding region 3. Importantly, the recruitment of MED1 and BRD4 was not obvious in CD4^+^ T cells of RA patients (Fig. 3B). To determine the molecular basis of the reduced MED1/BRD4 recruitment, we conducted in silico mining for the *UBASH3A* eRNA-coding regions and identified a MafK binding site. MafK heterodimerizes with BACH2, and this heterodimer is a silencing transcriptional factor, like Blimp1. Since BACH2 is highly expressed in lymphocytes, including B and T cells, we examined BACH2 and Blimp1, and the results showed that BACH2 was inversely recruited to *UBASH3A* regions 1 and 2 in CD4^+^ T cells of RA patients (Fig. 3B), suggesting lowered SE activity of these eRNA-coding SE regions. Based on these findings, we speculate the involvement of *UBASH3A*-SE dysfunction in UBASH3A downregulation in CD4^+^ T cells in RA patients. However, the molecular mechanism of the inverse recruitment of transcriptional regulators on the *UBASH3A*-coding SE regions remains unclear, and this issue requires further investigation.

The present study has several limitations. First, the study included a small number of UBASH3A-stained lymph node samples collected from patients with dermatomyositis as the control group for RA patients. Second, CD4^+^ T cells were collected from the peripheral blood of only a few subjects, which was probably insufficient to confirm the genetic association of the tested SNP (rs1893592) with the RA pathogenic process. Additional subjects are clearly required, not only regarding this issue, but also with regard to other assays. Thus, the in vivo role of UBASH3A in RA pathogenesis remains to be determined. However, the presence of high IL-6 levels in RA patients is widely known in clinical practice to be involved in the progression of the pathology, such as inflammation, immune abnormalities, and joint destruction [24]. Moreover, IL-6 antibody is therapeutically effective in suppressing RA pathogenesis [24]. Furthermore, analysis of collagen-induced arthritis in wild-type and *Ubash3a*-deficient mice showed significantly worse arthritis scores in the null mutants [25]. Given that the *UBASH3A* gene locus displays a SE architecture, the present findings suggest that the low expression levels of UBASH3A associated with SE dysfunction promote, at least in part, IL-6-associated RA pathogenesis. Further, stimulation of CD3/CD28 on CD4^+^ T cells in the absence of transfection with *UBASH3A* induced higher rates of IL-6 expression in RA than in HD. Given that UBASH3A is a suppressor of TCR signaling, the lower endogenous UBASH3A levels in CD4^+^ T cells from RA patients compared to HD seemed reasonable. We further found that *UBASH3A* was negatively correlated with the *IL-6* gene level in CD4^+^ T cells of RA patients. Thus, in agreement with previous studies [21], we believe that IL-6 increases in RA patients accompany UBASH3A downregulation, which enhances the persistence of inflammatory pathological state (Supplementary Figure 4).

## Conclusions

*UBASH3A* mRNA was downregulated in CD4^+^ T cells of RA patients. Furthermore, the recruitment of BACH2 and the lack of co-activators in CD4^+^ T cells reduced *UBASH3A* SE activity. Given that UBASH3A significantly attenuated IL-6 cytokine production in TCR signaling-activated CD4^+^ T cells, low levels of UBASH3A resulted in high levels of IL-6. These results suggest that UBASH3A is a potentially useful therapeutic target in RA.

## Supplementary Information

**Additional file 1.** All full-length images of Western blotting data. Uncropped full-length images of Figs. 1E and 4B are shown in the upper and lower spaces, respectively.

**Additional file 2.** The numbers shown here corresponds to ones of uncropped full-length images of Additional files 3, 4, 5.

**Additional file 3.** All full-length images of PCR data (#1). Uncropped full-length images of Fig. 3B are shown. The corresponding numbers are shown in Additional file 2.

**Additional file 4.** All full-length images of PCR data (#2). Uncropped full-length images of Fig. 3B are shown. The corresponding numbers are shown in Additional file 2.

**Additional file 5.** All full-length images of PCR data (#3). Uncropped full-length images of Fig. 3B are shown. The corresponding numbers are shown in Additional file 2.

**Additional file 6.** All full-length images of PCR data (#4). Uncropped full-length images of Fig. 6B are shown.

**Additional file 7: Supplementary Table S1.** Baseline characteristics of patients with RA. Data are expressed as mean ± SD. MS, morning stiffness; TJC, Tender Joint Count; SJC, Swollen Joint Count; PGA, patient's global assessment; EGA, evaluator's global assessment; HAQ, health assessment questionnaire; CRP, C-reactive protein; ESR, erythrocyte sedimentation rate; RF, rheumatoid factor; MMP-3, matrix metalloproteinase-3; KL-6, sialylated carbohydrate antigen KL-6; ACPA, anti-citrullinated protein antibody; CDAI, Clinical Disease Activity Index; SDAI, Simplified disease activity index; DAS28, Disease Activity Score; N.D., Not determined. **Supplementary Table S2.** LNAs, probes and primers used in the study. * phosphorothioate backbone Oligonucleotides used for LNA transfection (5’→3’ sequence), SNP-PCR(Life Technologies assay number), ChIP-PCR (5’→3’ sequence), qPCR (Life Technologies assay number). **Supplementary Figure S1.** Expression level of UBASH3A in different CD4^+^ T cell subsets. UBASH3A mRNA expression level was quantified in Th1, Th2, Th17, and regulatory T cells by RT-qPCR. The amount of UBASH3A transcript was expressed relative to that of GAPDH. Data are mean ± standard deviation of three independent experiments. *p<0.05, vs. Th1, by Dunnett's multiple comparison test. RQ, Relative quantification. **Supplementary Figure S2.** Expression levels of UBASH3A protein in CD4^-^ and CD4^+^ T cells of HD (n=3) and RA patients (n=3) evaluated by Western blotting. The results were quantified using ImageJ software. The amount of UBASH3A was normalized to that of β-actin used as a loading control. Three independent experiments were performed. **Supplementary Figure S3.** Quantification of UBASH3A protein expression in CD4^+^ T cells from the lymph nodes of dermatomyositis (DM) and RA patients. Lymph nodes were evaluated by immunofluorescence staining. The results were quantified using ImageJ software. The amount of UBASH3A (*, ** and *** in Fig. 1F) was normalized to that of CD4. Results were expressed as dot plots. RQ, Relative quantification. **Supplementary Figure S4.** Schematic diagram of the study findings. 1. Epigenetic dysfunction. Recruitment of BACH2 and MED1/BRD4 to UBASH3A gene is promoted and suppressed, respectively, in RA CD4^+^ T cells, but not the control. 2. Low levels of UBASH3A. Expression of UBASH3A gene is suppressed through the above mechanism. 3. Enhanced TCR signaling. UBASH3A confers weak ability for negative regulator of TCR signaling due to low levels of UBASH3A, resulting in enhanced TCR signaling. 4. IL-6 induction. TCR signaling activates IL-6 gene, leading to overproduction of IL-6 and sustained inflammation.

## Data Availability

Not applicable.

## Abbreviations

BACH2: BTB and CNC homology 2
BioGPS: Bio gene portal system
Blimp1: B lymphocyte-induced maturation protein-1
BRD4: Bromodomain-containing protein 4
ChIP: Chromatin immunoprecipitation
dbSUPER: Database of super-enhancer
eRNA: Enhancer RNA
gapdh: Glyceraldehyde-3-phosphate dehydrogenase
GWAS: Genome-wide association study
H3K27ac: Histone H3 containing the acetylated lysine 27
IL: Interleukin
LNA: Locked nucleic acid
MED1: Mediator of RNA polymerase II transcription subunit 1
NF-κB: Nuclear factor-kappa B
PBMCs: Peripheral blood mononuclear cells
RA: Rheumatoid arthritis
RQ: Relative quantification
SE: Super-enhancer
SNP: Single-nucleotide polymorphism
TCR: T cell receptor
TNF: Tumor necrosis factor
