# Correction: IL-6 production through repression of UBASH3A gene via epigenetic dysregulation of super-enhancer in CD4+ T cells in rheumatoid arthritis

**DOI:** 10.1186/s41232-022-00246-2

**Published:** 2022-12-02

**Authors:** Kaoru Yamagata, Shingo Nakayamada, Tong Zhang, Anh Phuong Nguyen, Naoaki Ohkubo, Shigeru Iwata, Shigeaki Kato, Yoshiya Tanaka

**Affiliations:** 1 The First Department of Internal Medicine, University of Occupational and Environmental Health, Japan, 1-1 Iseigaoka, Yahata-nishi, Kitakyushu, Fukuoka, 807-8555 Japan; 2 Graduate School of Life Science and Engineering, Iryo Sosei University, Iwaki, Fukushima 970-8551 Japan

**Correction: Inflamm Regen 42, 46 (2022)**

**https://doi.org/10.1186/s41232-022-00231-9**

Following publication of the original article [1], the authors reported that the fifth author’s name should be corrected from “Naoyuki Ohkubo” to “Naoaki Ohkubo”.

The correct author name has been provided in this Correction.

The original article [1] has been corrected.
