# Supplementary material for: IL-6 production through repression of UBASH3A gene via epigenetic dysregulation of super-enhancer in CD4+ T cells in rheumatoid arthritis
Source: Inflamm Regen. 2022 Nov 3;42:46. doi: 10.1186/s41232-022-00231-9 (PMC9632101; doi:10.1186/s41232-022-00231-9)

Additional file 1

All full-length images of Western blotting data. Uncropped full-length images of Figure 1E and Figure 4B are shown in the upper and lower spaces, respectively.

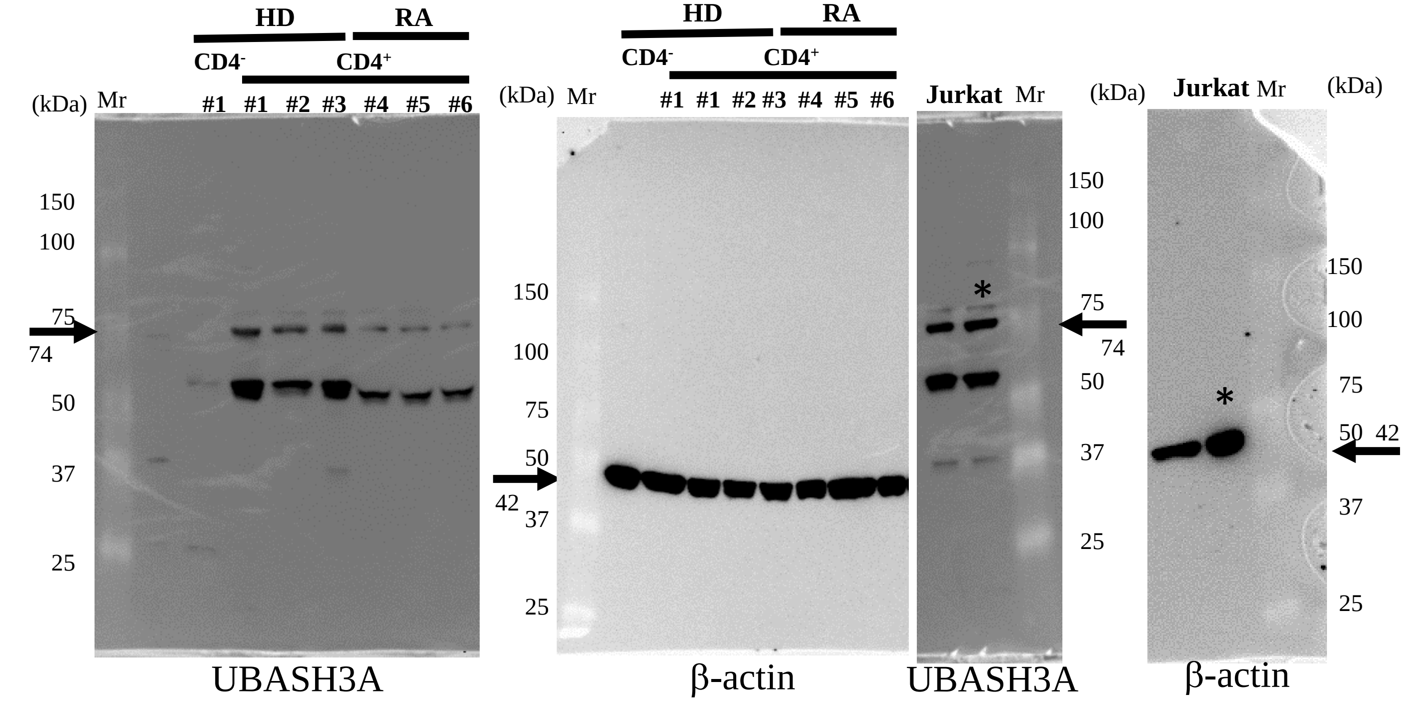

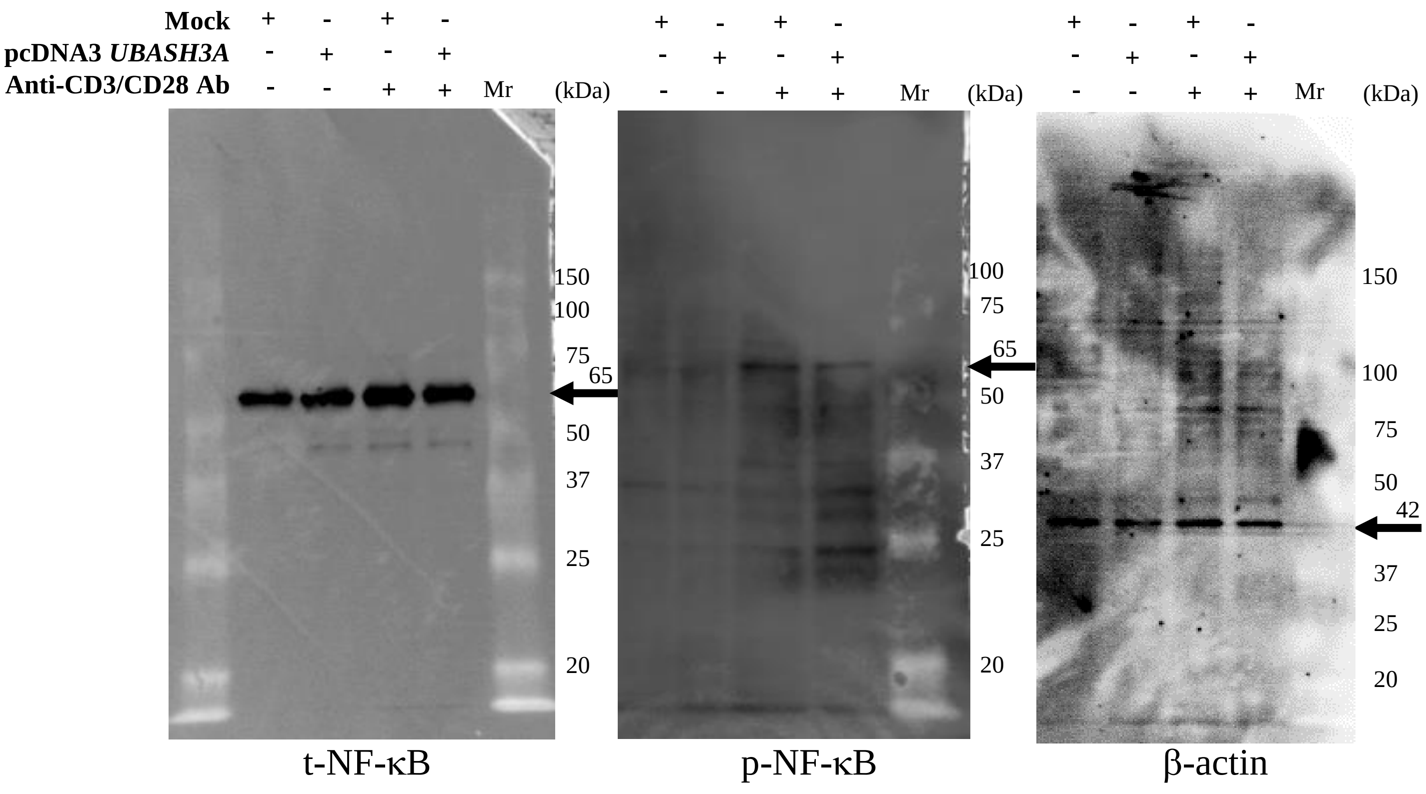

Supplement: Supplementary file 1 — Additional file 1. All full-length images of Western blotting data. Uncropped full-length images of Figs. 1E and 4B are shown in the upper and lower spaces, respectively. [file 41232_2022_231_MOESM1_ESM.pdf]
