# Supplementary material for: IL-6 production through repression of UBASH3A gene via epigenetic dysregulation of super-enhancer in CD4+ T cells in rheumatoid arthritis
Source: Inflamm Regen. 2022 Nov 3;42:46. doi: 10.1186/s41232-022-00231-9 (PMC9632101; doi:10.1186/s41232-022-00231-9)
Supplement: Supplementary file 2 — Additional file 2. The numbers shown here corresponds to ones of uncropped full-length images of Additional files 3, 4, 5. [file 41232_2022_231_MOESM2_ESM.pdf]

Additional file 2

The numbers shown here corresponds to ones of uncropped full-length images of Additional files 3-5.

	1	2	3	1	2	3
	HD_1 RA_1	HD_1 RA_1	HD_1 RA_1	HD_4 RA_4	HD_4 RA_4	HD_4 RA_4
Input	①	⑨	⑰	㉕	㉝	㊶
IgG	②	⑩	⑱	㉖	㉞	㊷
BACH2	③	⑪	⑲	㉗	㉟	㊸
Blimp1	④	⑫	⑳	㉘	㊱	㊹
H3K27ac	⑤	⑬	㉑	㉙	㊲	㊺
p300	⑥	⑭	㉒	㉚	㊳	㊻
MED1	⑦	⑮	㉓	㉛	㊴	㊼
BRD4	⑧	⑯	㉔	㉜	㊵	㊽
Size (bp)	106	108	117	106	108	117
