# Supplementary material for: IL-6 production through repression of UBASH3A gene via epigenetic dysregulation of super-enhancer in CD4+ T cells in rheumatoid arthritis
Source: Inflamm Regen. 2022 Nov 3;42:46. doi: 10.1186/s41232-022-00231-9 (PMC9632101; doi:10.1186/s41232-022-00231-9)

Additional file 3

All full-length images of PCR data (#1). Uncropped full-length images of Figure 3B are shown. The corresponding numbers are shown in Additional file 2.

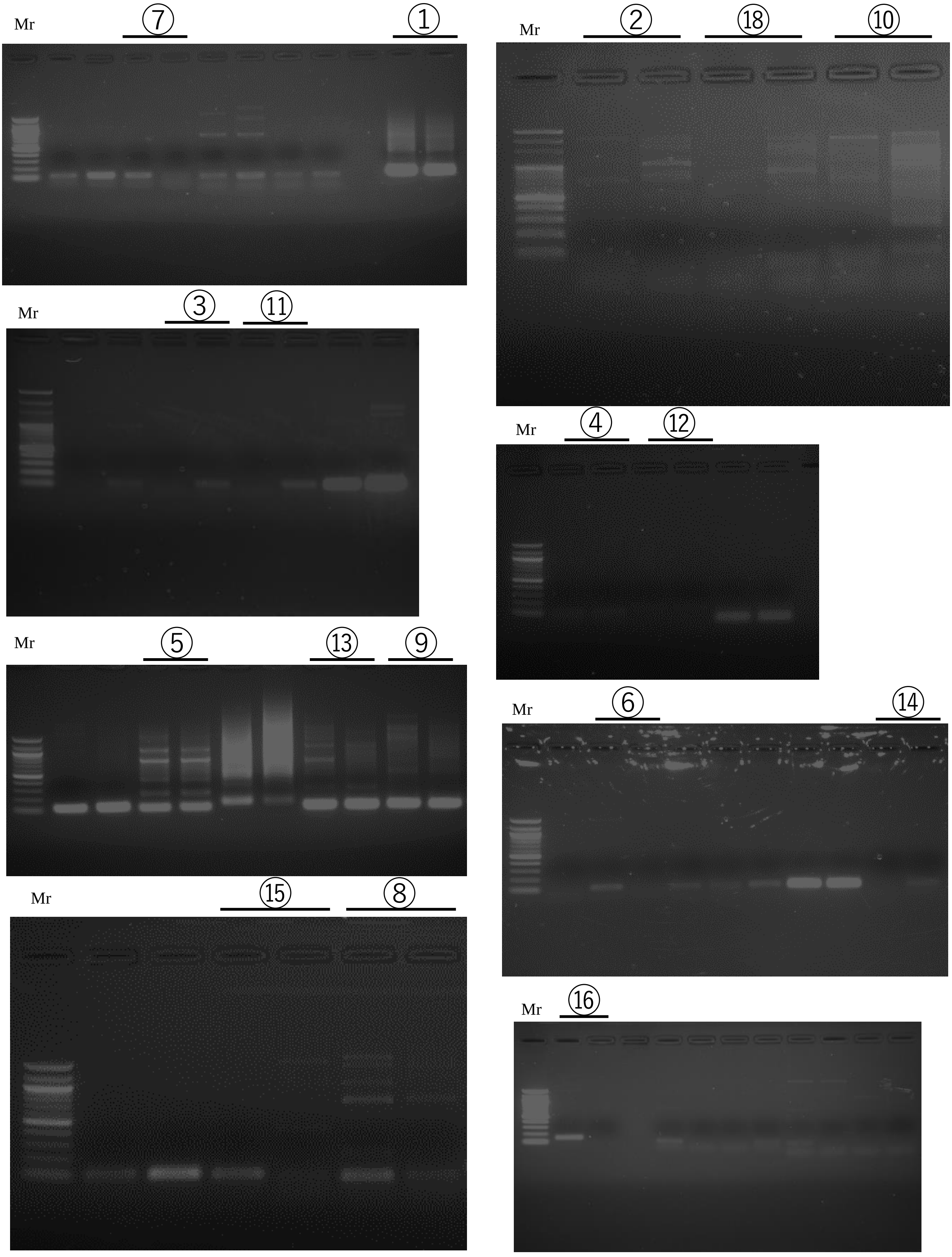

Supplement: Supplementary file 3 — Additional file 3. All full-length images of PCR data (#1). Uncropped full-length images of Fig. 3B are shown. The corresponding numbers are shown in Additional file 2. [file 41232_2022_231_MOESM3_ESM.pdf]
