# Supplementary material for: IL-6 production through repression of UBASH3A gene via epigenetic dysregulation of super-enhancer in CD4+ T cells in rheumatoid arthritis
Source: Inflamm Regen. 2022 Nov 3;42:46. doi: 10.1186/s41232-022-00231-9 (PMC9632101; doi:10.1186/s41232-022-00231-9)
Supplement: Supplementary file 7 — Additional file 7: Supplementary Table S1. Baseline characteristics of patients with RA. Data are expressed as mean ± SD. MS, morning stiffness; TJC, Tender Joint Count; SJC, Swollen Joint Count; PGA, patient's global assessment; EGA, evaluator's global assessment; HAQ, health assessment questionnaire; CRP, C-reactive protein; ESR, erythrocyte sedimentation rate; RF, rheumatoid factor; MMP-3, matrix metalloproteinase-3; KL-6, sialylated carbohydrate antigen KL-6; ACPA, anti-citrullinated protein antibody; CDAI, Clinical Disease Activity Index; SDAI, Simplified disease activity index; DAS28, Disease Activity Score; N.D., Not determined. Supplementary Table S2. LNAs, probes and primers used in the study. * phosphorothioate backbone Oligonucleotides used for LNA transfection (5’→3’ sequence), SNP-PCR(Life Technologies assay number), ChIP-PCR (5’→3’ sequence), qPCR (Life Technologies assay number). Supplementary Figure S1. Expression level of UBASH3A in different CD4+ T cell subsets. UBASH3A mRNA expression level was quantified in Th1, Th2, Th17, and regulatory T cells by RT-qPCR. The amount of UBASH3A transcript was expressed relative to that of GAPDH. Data are mean ± standard deviation of three independent experiments. *p<0.05, vs. Th1, by Dunnett's multiple comparison test. RQ, Relative quantification. Supplementary Figure S2. Expression levels of UBASH3A protein in CD4- and CD4+ T cells of HD (n=3) and RA patients (n=3) evaluated by Western blotting. The results were quantified using ImageJ software. The amount of UBASH3A was normalized to that of β-actin used as a loading control. Three independent experiments were performed. Supplementary Figure S3. Quantification of UBASH3A protein expression in CD4+ T cells from the lymph nodes of dermatomyositis (DM) and RA patients. Lymph nodes were evaluated by immunofluorescence staining. The results were quantified using ImageJ software. The amount of UBASH3A (*, ** and *** in Fig. 1F) was normalized to that of CD4. Results [file 41232_2022_231_MOESM7_ESM.docx]

**Supplementary information**

**Supplementary figures**

**
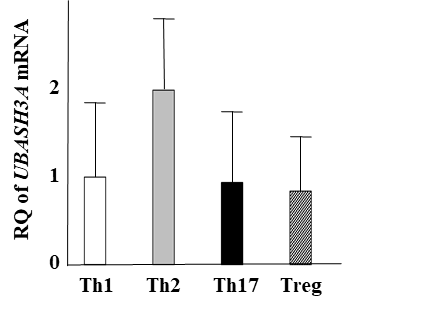
**

**Supplementary Figure S1. Expression level of UBASH3A in different CD4+ T cell subsets.** *UBASH3A* mRNA expression level was quantified in Th1, Th2, Th17, and regulatory T cells by RT-qPCR. The amount of *UBASH3A* transcript was expressed relative to that of *GAPDH*. Data are mean ± standard deviation of three independent experiments. *p<0.05, vs. Th1, by Dunnett's multiple comparison test. RQ, Relative quantification.

**
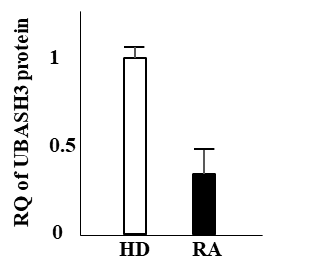
**

**Supplementary Figure S2. Expression levels of UBASH3A protein in CD4^−^ and CD4^+^ T cells of HD (n=3) and RA patients (n=3) evaluated by Western blotting.** The results were quantified using ImageJ software. The amount of UBASH3A was normalized to that of β-actin used as a loading control. Three independent experiments were performed.

**
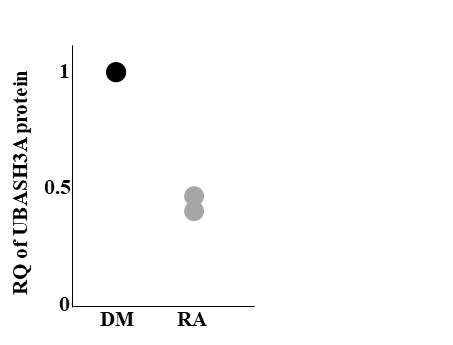
**

**Supplementary Figure S3. Quantification of UBASH3A protein expression in CD4^+^ T cells from the lymph nodes of dermatomyositis (DM) and RA patients.** Lymph nodes were evaluated by immunofluorescence staining. The results were quantified using ImageJ software. The amount of UBASH3A (*, ** and *** in Figure 1F) was normalized to that of CD4. Results were expressed as dot plots. RQ, Relative quantification.

**
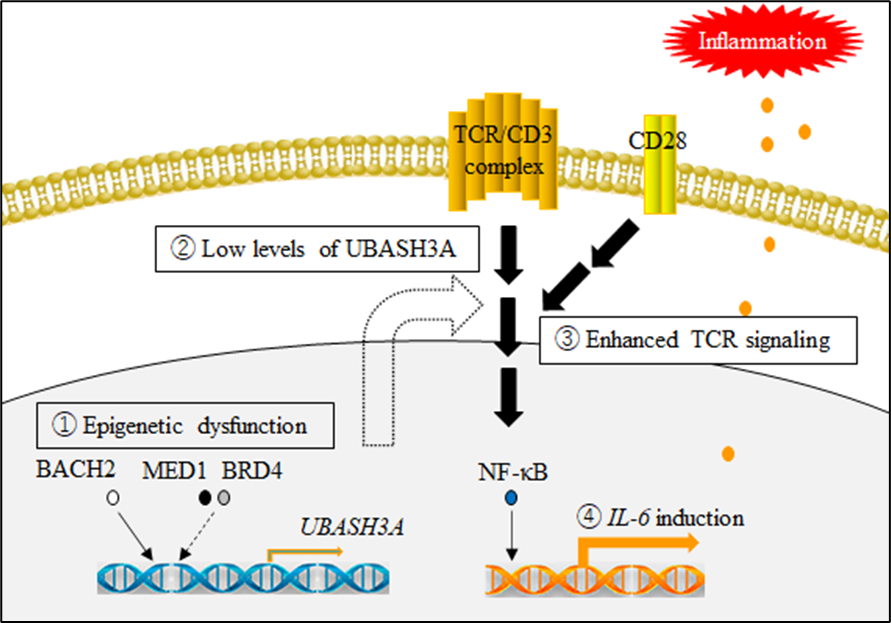
**

**Supplementary Figure S4. Schematic diagram of the study findings.** 1. Epigenetic dysfunction. Recruitment of BACH2 and MED1/BRD4 to UBASH3A gene is promoted and suppressed, respectively, in RA CD4^+^ T cells, but not the control. 2. Low levels of UBASH3A. Expression of *UBASH3A* gene is suppressed through the above mechanism. 3. Enhanced TCR signaling. UBASH3A confers weak ability for negative regulator of TCR signaling due to low levels of UBASH3A, resulting in enhanced TCR signaling. 4. IL-6 induction. TCR signaling activates *IL-6* gene, leading to overproduction of IL-6 and sustained inflammation.

**Supplementary tables**

|  | RA (n=24) | HD (n=11) | p-value |
| --- | --- | --- | --- |
| Age | 57.6±12.6 | 50.5±7.0 | 0.05956 |
| Sex (M:F) | (8:16) | (3:8) | 0.71123 |
| Disease duration (month) | 80.9±108.0 | N.D. |  |
| MS (min) | 179.4±97.5 | N.D. |  |
| TJC | 9.1±6.1 | N.D. |  |
| SJC | 9.0±5.6 | N.D. |  |
| PGA (mm) | 60.9±27.1 | N.D. |  |
| EGA (mm) | 57.5±17.8 | N.D. |  |
| HAQ | 1.4±0.8 | N.D. |  |
| CRP (mg/ml) | 3.9±4.8 | N.D. |  |
| ESR (mm/h) | 66.1±29.9 | N.D. |  |
| RF (U/ml) | 137±135 | N.D. |  |
| MMP-3 (ng/ml) | 295±298 | N.D. |  |
| KL-6 (U/ml) | 269±150 | N.D. |  |
| ACPA (U/ml) | 422±867 | N.D. |  |
| CDAI | 136±49 | N.D. |  |
| SDAI | 140±51 | N.D. |  |
| DAS28-ESR | 6.1±1.2 | N.D. |  |

**Supplementary Table S1. Baseline characteristics of patients with RA.** Data are expressed as mean ± SD. MS, morning stiffness; TJC, Tender Joint Count; SJC, Swollen Joint Count; PGA, patient's global assessment; EGA, evaluator's global assessment; HAQ, health assessment questionnaire; CRP, C-reactive protein; ESR, erythrocyte sedimentation rate; RF, rheumatoid factor; MMP-3, matrix metalloproteinase-3; KL-6, sialylated carbohydrate antigen KL-6; ACPA, anti-citrullinated protein antibody; CDAI, Clinical Disease Activity Index; SDAI, Simplified disease activity index; DAS28, Disease Activity Score; N.D., Not determined.

| **Antisense LNA GapmeR** | |
| --- | --- |
| eRNA 1 sense | C*C*A*G*A*A*G*C*A*T*A*G*A*A*T*A |
| eRNA 1 antisense | A*T*G*A*T*T*G*G*G*T*G*T*A*A*A*C |
| eRNA 2 sense | G*A*A*G*G*C*A*A*G*T*G*A*T*A*G*T |
| eRNA 2 antisense | T*G*G*T*C*A*A*G*G*T*C*T*T*A*G*G |
| eRNA 3 sense | C*T*C*T*G*G*T*G*T*C*C*T*C*T*T*T |
| eRNA 3 antisense | C*C*T*T*A*A*A*G*A*G*T*C*T*A*G*A |
| Negative control B | G*C*T*C*C*C*T*T*C*A*A*T*C*C*A*A |
|  |  |
| **SYBR Green PCR** |  |
| eRNA 1 sense | CAG AGG ACC CCA GTT CAG TG |
| eRNA 1 antisense | ATT TCC GTC TCT GCC CTT TT |
| eRNA 2 sense | GGT GTG GAG CCT CTG ATT CT |
| eRNA 2 antisense | GAC CTA CAC TTG CGG GTG AC |
| eRNA 3 sense | AAA GAG CCA GGA AAG AGG ACA |
| eRNA 3 antisense | TCT TTT CCT CAG CCA CTT CC |
| gapdh sense | GTC TCC TCT GAC TTC AAC AGC G |
| gapdh antisense | ACC CTG TTG CTG TAG CCA A |
|  |  |
| **SNP-PCR** | C___3270998_1_ |
|  |  |
| **ChIP-PCR** |  |
| ubash3a FW1 | GCC TGG ACC TGC CAT AGT T |
| ubash3a RV1 | CCA GGA AGG GCA TCT TTT CT |
| ubash3a FW2 | AGC ACT TGC TGA GTG CAG TG |
| ubash3a RV2 | CTG GGA CCA TCT CCT C |
| ubash3a FW3 | TCA CTT TGG TTT CTG CCA CT |
| ubash3a RV3 | GCA GTA AAA GCC CCA GTG AG |
| IL-6 FW1 | CGT TTT TGG AGC AAG GTA GA |
| IL-6 RV1 | ATG CTT CTG GGG TCA AGA AA |
| IL-6 FW2 | GCC TCA ATG ACG ACC TAA GC |
| IL-6 RV2 | ACT CAT GGG AAA ATC CCA CA |
|  |  |
| **qPCR** |  |
| UBASH3A | Hs00955173_m1 |
| IL1B | Hs01555410_m1 |
| IL6 | Hs00174131_m1 |
| IL17A | Hs00174383_m1 |
| TNF | Hs00174128_m1 |
| GAPDH | Hs99999905_m1 |

**Supplementary Table S2. LNAs, probes and primers used in the study.** * phosphorothioate backbone Oligonucleotides used for LNA transfection (5’→3’ sequence), SNP-PCR(Life Technologies assay number), ChIP-PCR (5’→3’ sequence), qPCR (Life Technologies assay number).
